# CPR-induced consciousness in hypothermic cardiac arrest: Where is the limit of tolerance of the human brain? A case report

**DOI:** 10.1186/s13049-025-01426-y

**Published:** 2025-07-01

**Authors:** Łukasz Migiel, Tomasz Darocha, Hubert Hymczak, Paweł Podsiadło, Konrad Mendrala, Sylweriusz Kosiński

**Affiliations:** 1 Tatra Mountain Rescue Service, Zakopane, Poland; 2 Department of Anaesthesiology and Intensive Care, Medical University of Silesia, Katowice, Poland; 3 Department of Anaesthesiology and Intensive Care, Andrzej Frycz Modrzewski Krakow University, Krakow, Poland; 4 Department of Emergency Medicine, Jan Kochanowski University, Kielce, Poland; 5 Mountain Medicine Laboratory, Jagiellonian University Medical College, Zakopane, Poland

**Keywords:** Hypothermia, accidental, Cardiac arrest, sudden, Resuscitation, Consciousness

## Abstract

**Background:**

CPR-induced consciousness (CPRIC) is defined as consciousness during CPR, ranging from eye opening to combative behaviour and vocalisation, despite the absence of spontaneous circulation. CPRIC has not previously been reported in hypothermic cardiac arrest.

**Patient presentation:**

A middle-aged man who was pulled from cold water appeared conscious during CPR, despite confirmed cardiac arrest and severe accidental hypothermia. An additional factor that could have influenced the victim’s behaviour was severe hypoglycaemia. The patient was rewarmed with veno-arterial extracorporeal membrane oxygenation (V-A ECMO) and discharged from the hospital without any neurological deficits.

**Conclusions:**

In hypothermic cardiac arrest, the paradoxical preservation of consciousness may be a consequence of adequate cerebral perfusion during cardiopulmonary resuscitation and the neuroprotective effect of hypothermia, despite other risk factors for brain injury.

**Supplementary Information:**

The online version contains supplementary material available at 10.1186/s13049-025-01426-y.

## Background

Cardiac arrest is a significant source of mortality, but prompt initiation of cardiopulmonary resuscitation (CPR) offers potential reversibility. CPR can limit cellular hypoxia until cardiac, pulmonary, and cerebral function is restored with the return of spontaneous circulation (ROSC). During this period, although oxygen delivery to the brain is critically low, some victims may exhibit realistic behaviors suggestive of preserved consciousness of as yet unexplained etiology [1–3]. The coexistence of other factors with cardiac arrest that may be either neuroprotective or devastating creates an intriguing clinical scenario that opens the door to discussion of the tolerance of the human brain. We present a case of preserved consciousness induced by CPR (CPRIC) in a patient with hypothermic cardiac arrest (HCA) associated with severe hypoglycemia. To our knowledge, this is the first documented case of visible signs of consciousness occurring despite the coexistence of three factors that, at least in theory, should preclude the possibility of preserved consciousness.

### Case presentation

**Fig. 1:**
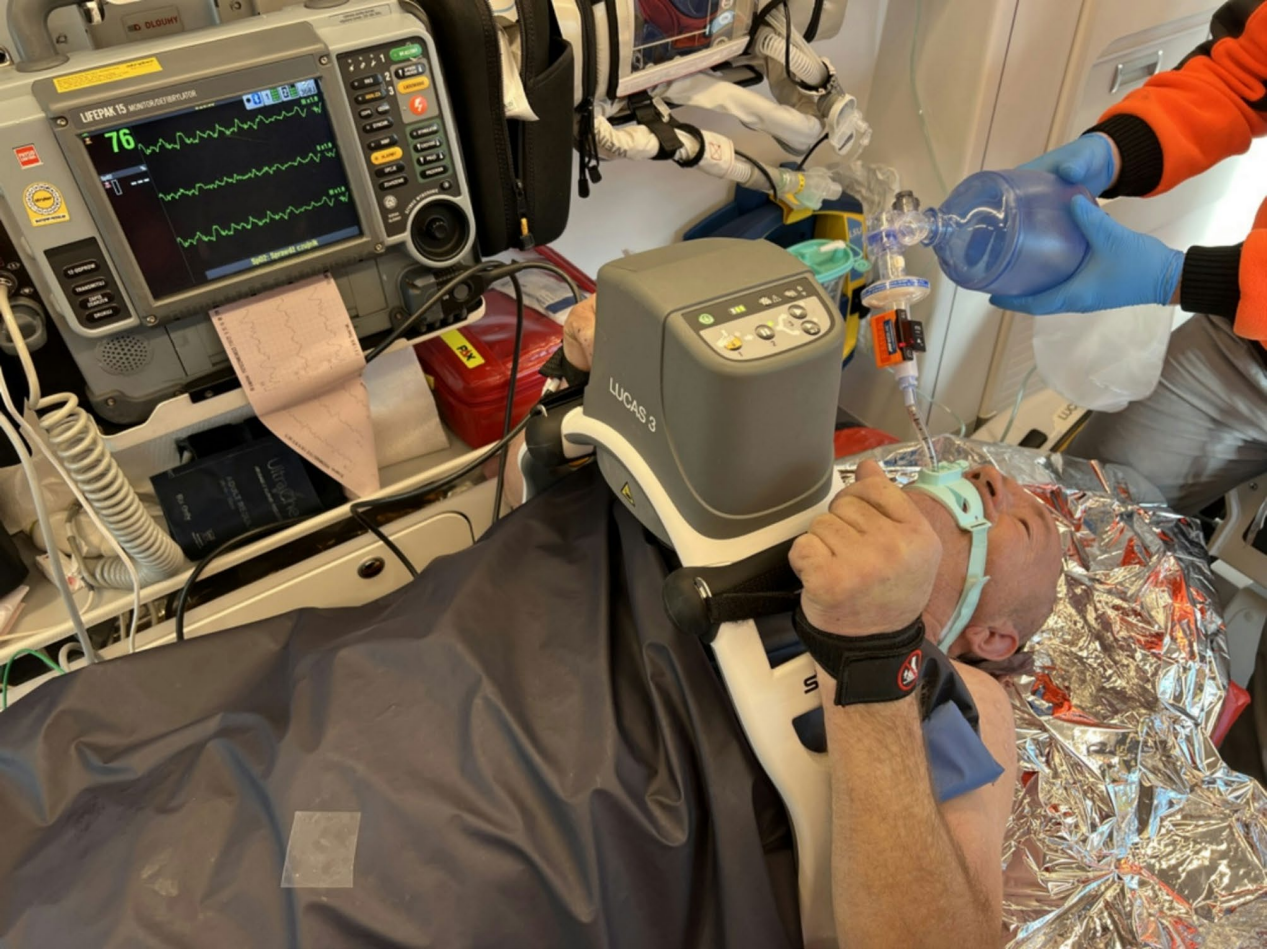
Resuscitation procedures during ambulance transport

The patient was a 57-year-old man found by police officers at approximately 8 a.m. on a November morning. The air temperature was approximately 0 °C, and the victim lay supine in the shallows of a mountain river, with the face above the water surface. It was not determined how he fell into the river or how long he had been there. The police pulled him to the shore and called an ambulance. The man was moving and gesticating, clearly cold to the touch, stiff, and uttering incomprehensible sounds. The ambulance arrived at the scene at 8:28 a.m. The paramedics found no vital signs during an extended period of examination and began cardiopulmonary resuscitation with manual chest compressions. Multifunctional electrodes were applied, ventricular fibrillation (VF) was detected, and a single shock with 360 Joules was delivered. The victim was intubated endotracheally on the first attempt via direct laryngoscopy. Neither sedatives nor muscle relaxants were administered. Ventilation was initiated with a self-inflating bag with 100% oxygen, and end-tidal CO2 (EtCO2) was measured at 12–14 mmHg. The VF persisted through the next two cycles of CPR, despite two additional defibrillation attempts. The patient was placed in an ambulance, his wet clothes were removed, and he was wrapped in a sleeping bag. The temperature measured at the tympanic membrane was 24.2 °C. The dispatch center was notified of the situation, prompting the initiation of the protocol for hypothermic circulatory arrest and the arrangement of direct transport to the nearest ECMO center located 95 km away. Owing to fog, the HEMS helicopter was unable to reach the scene. The ambulance set off to the designated patient handover location. During transport, chest compressions were performed continuously, and ventilation was provided with self-inflating bag at a rate of 8–10 per minute (Fig. 1). During CPR, the victim tried to stand, moved his hands, opened his eyes, and focused on the rescuers. When asked if he could hear, he nodded his head and tried to vocalise and remove the endotracheal tube. Resuscitation procedures were interrupted several times, and ROSC was suspected; however, the monitor revealed continuous VF, and no pulse was detected on the carotid artery. Point-of-care echocardiography confirmed ventricular fibrillation (Additional File). At 09:38 a.m., the patient was transferred to the HEMS helicopter, where mechanical chest compressions were continued. Owing to his persistent combativeness, groaning, and eye opening, a single dose of 100 mg ketamine was administered intravenously. However, the effect was short-lived and weak. During the flight, the doses were repeated twice. At 10:24 a.m., the patient was admitted to the ECMO center. Ventricular fibrillation was confirmed, and his esophageal temperature was 24.6 °C. His first temperature-corrected arterial blood gas analysis revealed a pH of 7.06, a PaO2 of 100 mmHg, a PaCO2 of 41 mmHg, a bicarbonate ion concentration of 11.2 mmol/L, a base deficit of 19.5 mmol/L, a lactate concentration of 9.5 mmol/L, a potassium concentration of 3.7 mmol/L, a hemoglobin concentration of 16.3 g/dL and a glucose concentration of 1.6 mmol/L. At 11:10 a.m., extracorporeal cardiopulmonary resuscitation (eCPR) was started. At 12:07, with a core temperature of 28.5 °C, a single defibrillation with 360 joules restored circulation with a regular heart rate of 75/min. Veno-arterial extracorporeal membrane oxygenation (V-A ECMO) was discontinued the same day, and the patient was extubated. His medical history was unremarkable, and he denied taking any medications. He had consumed alcohol the previous day and had only fragmentary memories of returning home. However, the patient was unable to recall any images from the resuscitation period or the hospital stay. On the fourth day, the patient was discharged from the intensive care unit with a cerebral performance category (CPC) score of 1. At the 12-month follow-up, his condition did not raise any concerns.

## Discussion

Current CPR techniques are designed to maintain oxygen delivery to tissues until spontaneous circulation can be restored. In addition to achieving ROSC, these techniques primarily serve to maintain cerebral blood flow, so the term cardiocerebral resuscitation (CCR) is commonly used. During cardiopulmonary resuscitation, there have been reports of realistic life-like behavior in pulseless patients receiving chest compressions [1–3]. Both visible signs of consciousness, such as combativeness, groaning, eye opening or the perception of lucidity, visual or auditory awareness, and near-death experiences, are reported. Interestingly, patients with awareness or recall of events do not always present with visible signs of consciousness [4].

The clinical manifestation of accidental hypothermia typically involves a progressive deterioration from mild neurologic impairment to cardiac instability and eventually loss of vital signs. Cerebrovascular autoregulation is lost at approximately 25 °C, and consciousness is commonly lost below 30 °C [5]. However, the level of consciousness may vary widely among patients at a given core temperature [6–8]. Clinical observations, supported by a retrospective analysis of patients with accidental hypothermia, have demonstrated a linear relationship between core temperature and decreased level of consciousness. The mean decrease in a vital sign associated with a 1 °C decrease in temperature was estimated to be 0.88 for the GCS (i.e., for 24 °C, the GCS would be 4–5) [9]. However, the response to cooling is highly individual. Some patients may exhibit only slightly decreased consciousness despite severe hypothermia. In a recent study on hypothermic cardiac arrest, one-third of patients who sustained HCA (all with a core temperature ≤ 28 °C) were reported as “not unconscious” at the initial assessment [10].

Instances of CPR-induced consciousness appear to be more common in cardiac arrests witnessed by professional rescuers, particularly those caused by shockable rhythms with presumed cardiac etiology. The use of mechanical devices may be more frequently associated with consciousness during CPR than traditional chest compressions [11]. The cause of CPR-induced consciousness remains unclear but is likely the result of a combination of factors, including high-quality CPR, circumstances (witnessed cardiac arrest, skilled medical personnel) and individual factors, such as preserved cerebral autoregulation, a higher ischemic threshold, or the presence of comorbidities influencing brain oxygenation and metabolism [2, 11].

One of the additional factors that could influence a patient’s condition and prognosis is hypoglycaemia. In the setting of cellular stress due to critically low cerebral blood flow during CPR, the hypothalamic‒pituitary‒adrenal axis stimulates gluconeogenesis and glycogenolysis, providing energy substrates to maintain cellular integrity. Failure to increase the blood glucose concentration in response to CPR appears to be a poor prognostic indicator in normothermic cardiac arrest patients. However, in patients with hypothermic cardiac arrest, hypoglycaemia prior to eCPR may not preclude survival with good neurological outcome. Neither the tolerance of the human brain to hypoglycaemia in hypothermia nor the lowest tolerated glucose concentration in relation to the core temperature has been established.

Despite the increasing number of reports of CPRIC, there is a lack of standardised guidelines for its recognition and treatment, including the potential need for sedation. CPRIC poses ethical dilemmas regarding the administration of anaesthetics given the risk of haemodynamic compromise. Including information about CPRIC in resuscitation training programs may better prepare healthcare providers for this unusual situation. Furthermore, investigating whether the CPRIC can predict favourable neurologic outcomes after CPR is highly warranted.

Current scientific views regarding the origin of consciousness vary widely and range from an effect arising from neuronal networks or neuronal quantum processes to a separate undiscovered scientific entity [12, 13]. It would be interesting to explain the underlying mechanism of preserved consciousness in a setting where two or even three strong factors come together, each of which separately causes a profound impairment of cerebral blood flow and cerebral function. Perhaps an underlying neuroprotective effect of hypothermia in combination with selective adaptive changes in cerebral blood flow may have had some significance. We hope that our case report will point to new directions for further research into this phenomenon.

## Conclusion

CPR-induced consciousness is a rare and poorly understood phenomenon. In hypothermic cardiac arrest, the paradoxical preservation of consciousness may be a consequence of adequate cerebral perfusion during cardiopulmonary resuscitation and the neuroprotective effect of hypothermia, despite other risk factors for brain injury.

## Electronic supplementary material

Below is the link to the electronic supplementary material.

Supplementary Material 1

## Data Availability

No datasets were generated or analysed during the current study.

## Abbreviations

CCR: Cardiocerebral resuscitation
CPC: Cerebral performance category
CPR: Cardiopulmonary resuscitation
CPRIC: Cardiopulmonary resuscitation-induced consciousness
eCPR: Extracorporeal cardiopulmonary resuscitation
ETCO2: End tidal carbon dioxide
GCS: Glasgow Coma Scale
HCA: Hypothermic cardiac arrest
HEMS: Helicopter emergency medical system
ROSC: Return of spontaneous circulation
V-A ECMO: Veno-arterial extracorporeal membrane oxygenation
VF: Ventricular fibrillation
